# The Role of *TcCYP6K1* and *TcCYP9F2* Influences Trehalose Metabolism under High-CO_2_ Stress in *Tribolium castaneum* (Coleoptera)

**DOI:** 10.3390/insects15070502

**Published:** 2024-07-05

**Authors:** Liwen Guan, Xianzhong Wang, Sijing Wan, Yuanyuan Wang, Xinyu Zhang, Shigui Wang, Can Li, Bin Tang

**Affiliations:** 1 College of Life and Environmental Sciences, Hangzhou Normal University, Hangzhou 311121, China; guanliwen1010@163.com (L.G.); wangxz9264@163.com (X.W.); wsjw9898@163.com (S.W.); sgwang@hznu.edu.cn (S.W.); 2 Key Laboratory of Surveillance and Management of Invasive Alien Species in Guizhou Education Department, Department of Biology and Engineering of Environment, Guiyang University, Guiyang 550005, China; wyy5211234@163.com (Y.W.); zhxy0829@163.com (X.Z.)

**Keywords:** *Tribolium castaneum*, cytochrome P450, CO_2_ stress, trehalose metabolism, RNAi

## Abstract

**Simple Summary:**

Due to issues with pesticide residues and resistance caused by traditional pesticides, the use of modified atmosphere storage technology has become increasingly popular. However, research has shown that prolonged exposure to high concentrations of CO_2_ for pest control in this technology can lead to insect resistance to hypoxia. Therefore, it is necessary to find out the resistance mechanism. This study identifies *TcCYP6K1* and *TcCYP9F2* as key factors in the response to high CO_2_ in *Tribolium castaneum*, suggesting that these genes may affect the resistance to high CO_2_ by influencing in the synthesis or breakdown of the carbohydrate metabolism pathways. These findings provide a theoretical basis for the combined use of novel nucleic acid pesticides and modified atmosphere treatment.

**Abstract:**

Cytochrome P450 monooxygenases (CYP), crucial detoxification enzymes in insects, are involved in the metabolism of endogenous substances as well as the activation and degradation of exogenous compounds. In this study, *T. castaneum* was utilized to investigate the roles of *TcCYP6K1* and *TcCYP9F2* genes influencing in the trehalose metabolism pathway under high-CO_2_ stress. By predicting the functional sequences of *TcCYP6K1* and *TcCYP9F2* genes and analyzing their spatiotemporal expression patterns, it was discovered that both genes belong to the CYP3 group and exhibit high expression levels during the larval stage, decreasing during the pupal stage, while showing high expression in the fatty body, intestine, and malpighian tubules. Furthermore, following the knockdown of *TcCYP6K1* and *TcCYP9F2* genes in combination with treating larvae with 75% CO_2_, it was observed that larval mortality increased, and glycogen content significantly decreased, while trehalose content increased significantly. Additionally, membrane-bound trehalase enzyme activity declined, TPS gene expression was significantly upregulated, GS gene expression was significantly downregulated, and ATP content showed a marked decrease. In conclusion, CYP genes are critical responsive genes of *T. castaneum* to high CO_2_ levels, potentially impacting the insect’s resistance to carbon dioxide through their involvement in the synthesis or breakdown of the carbohydrate metabolism pathway. These findings could serve as a theoretical basis for the utilization of novel pesticides in low-oxygen grain storage techniques and offer new insights for environmentally friendly pest control strategies in grain storage.

## 1. Introduction

Food security is of great significance for maintaining the stability and development of the world, but in the food storage stage, the activities of storage pests will cause huge losses to stored food [1]. *Tribolium castaneum* is one of the most harmful stored grain pests [2,3]. Chemical fumigants such as phosphine (PH_3_) and sulfuryl fluoride (SO_2_F_2_, SF) are usually used to control storage pests in agricultural production. However, long-term use will not only cause a pesticide residue problem [4], but also induce insects to develop certain resistance [5]. For example, it has been observed that phosphine resistance exists in *T. castaneum* [6,7].

Therefore, the safe and environmentally friendly modified atmosphere storage technology has been increasingly recognized by the public [8]. Within a confined space, CO_2_ or N_2_ is artificially injected to increase the concentration of other gasses and reduce the oxygen content. This process induces the production of harmful metabolites by cells, causing damage and inhibiting the physiological activities of pests, ultimately leading to their death [9]. It is worth noting that the insecticidal mechanisms of N_2_ and CO_2_ atmospheres differ. N_2_ is non-toxic to pests, primarily causing pest suffocation due to oxygen deprivation. In addition to replacing O_2_, CO_2_ itself also poses a threat to stored grain pests [8]. In *Oryzaephilus surinamensis*, treatment with 96% N_2_ for 5 days shows a similar inhibitory effect on the weevil to the control effect with 10% CO_2_ [10]. High CO_2_ can suppress the genes encoding components of the mitochondrial electron transport chain in the pest’s body [11], leading to an increase in carbonic acid content and causing acidosis along with lactic acid in the pest’s body, resulting in oxidative damage to stored grain pests [12,13]. However, continuous use of controlled atmosphere (CA) technology can lead to pests developing resistance under high CO_2_ levels. Pests adapt to this environment by adjusting their physiological functions or altering gene expression levels internally, which poses a challenge to pest control through CA [14,15,16,17]. Therefore, it is essential to investigate insects’ protective mechanisms in a controlled atmosphere to facilitate the development of new integrated pest control approaches combining methods such as RNA insecticides, essential oils, and CA.

Cytochrome P450 monooxygenases (CYP) are important detoxification enzymes for insects. They metabolize external substances like pesticides, plant secondary metabolites, and other toxic compounds [18,19,20,21], as well as participate in the metabolism of endogenous substances such as hormones, steroids, and fatty acids [22,23]. Within the four gene clusters of the CYP gene family [24], CYP2 and Mito genes primarily regulate insect growth and development, including the biosynthesis of ecdysteroids. On the other hand, CYP3 and CYP4 genes are involved in responding to external environmental stimuli and are susceptible to the selective pressure of toxic substances present in the environment, such as insecticides and host metabolites [25,26]. The CYP3 family is a major group of insects’ CYPs, including subfamilies CYP3, CYP5, CYP6, CYP9, CYP28, and CYP308-301 [27]. The CYP6 family is unique to insects, with the closest relationship to the CYP9 family [28]. Generally, they are associated with the detoxification of toxic metabolites in members of the Diptera and Lepidoptera orders [29,30,31,32]. In *Helicoverpa armigera*, *CYP6AE14* is involved in the metabolism of plant toxins (gossypol) [33], while in *T. castaneum*, the *TcCYP6BQ8* gene plays a crucial role in the breakdown metabolism of terpinen-4-ol [34]. Furthermore, current research on the function of CYP primarily focuses on its involvement in detoxifying pesticides and other toxic substances. Previous studies have shown that the bZIP cap ‘n’ collar isoform C (CncC) can participate in resistance to Eugenol by regulating the expression of the P450 gene in insects [35]. But studies on the involvement of CYP genes in the detoxification of endogenous metabolites under hypoxic stress have only been reported in *Caenorhabditis elegans* and human tumor cells [36,37]. However, the specific role of CYP genes in regulating the endogenous detoxification mechanism of grain storage pests under low-oxygen stress remains unclear. Therefore, the development of a novel double-stranded RNA (dsRNA) pesticide targeting CYP for hypoxic stress shows practicality.

In our previous works, we conducted analyses on *T. castaneum* under different concentrations of CO_2_ stress using transcriptomics and metabolomics [38,39]. Through this analysis, we identified P450 genes that were significantly differentially expressed under high concentrations of CO_2_ stress, including *TcCYP6K1* (Gene ID: 658029) and *TcCYP9F2* (Gene ID: 664302). In this study, we knocked down the CYP genes in *T. castaneum* and investigated the role of CYP genes influencing in the trehalose metabolism pathway under high CO_2_ stress-induced physiological changes. This study aimed to explore the potential molecular targets *TcCYP6K1* and *TcCYP9F2* to reveal the molecular mechanisms of CYP in *T. castaneum* adaptation to high CO_2_ stress. This research provides a theoretical basis for the application of novel dsRNA pesticides in low-oxygen grain storage technology and offers new insights and targets for the sustainable and effective control of stored grain pests.

## 2. Materials and Methods

### 2.1. Insect Source and Feeding Method

The *T. castaneum* in this experiment was sourced from the national grain reserve in Xingyi, Guizhou. The insects were raised in a laboratory long-term, fed on flour, and kept under controlled conditions with a temperature of 28 ± 2 °C and a relative humidity of 65% ± 5% RH.

### 2.2. Bioinformatics Analysis

To analyze the target gene, several bioinformatic tools were utilized. Firstly, the BLAST comparison of the gene was conducted using the NCBI website (http://www.ncbi.nlm.nih.gov, accessed on 23 October 2023) to identify homologous sequences. Next, the amino acid structural domains were predicted using HMMER (https://www.ebi.ac.uk/Tools/hmmer, accessed on 2 February 2024). Subsequently, ExPASy ProtParam, an online analysis tool, was employed to assess the physicochemical properties of the encoded protein, such as amino acid composition, molecular weight, theoretical pI, and so on. The signal peptide of the sequence was then analyzed using SignalP 4.1 Server online software, focusing on C, Y, and S scores, as well as the mean S score value. Furthermore, transmembrane structures were analyzed with the TMHMM Server v. 2.0 online tool. Lastly, the phylogenetic tree was constructed using MEGA 11.0 software to elucidate the evolutionary relationships among the analyzed sequences [40,41].

### 2.3. Collection of Tissue and Developmental Expression Samples

Different life forms of *T castaneum* were selected as developmental expression samples, including larvae at 1–8 instars, pupae at 1–4 days, adults at 1 day, adults at 5 days, adults at 10 days, adults at 15 days, adults at 21 days, and adults at 30 days. Three biological replicates were established for each developmental stage, with each replicate consisting of 35 insects. Tissue expression samples were obtained from 8th-instar larvae of *T. castaneum*; dissected into the midgut, head, epidermis, fatty body, malpighian tubules, and wings under a Leica EZ4 HD stereo microscope (Leica, Wetzlar, Germany); and preserved in RNAiso Plus (Code No. 9109, Takara, Kyoto, Japan). Three biological replicates were set up, with each replicate dissecting 100 larvae. All samples were flash-frozen in liquid nitrogen and stored at −80 °C in an ultra-low-temperature freezer for the later detection of changes in *TcCYP6K1* (TcasGA2_TC032509) and *TcCYP9F2* (TcasGA2_TC006445) expression levels.

### 2.4. RNA Extraction and cDNA Synthesis

Total RNA was extracted from the experimental insect samples using RNAiso Plus. The purity and concentration of the extracted RNA were analyzed by measuring 1 µL of RNA with the NanoDrop 2000 spectrophotometer (Thermo Fisher Scientific, Waltham, MA, USA). The integrity of the extracted RNA was verified by analyzing 2–3 µL of RNA through 1% agarose gel electrophoresis. Any remaining RNA was stored at −80 °C for future experiments. Subsequently, synthesis of the first-strand cDNA was performed following the instructions of the PrimeScript^®^ RT Reagent Kit With gDNA Eraser (Code No. RR047A, Takara, Kyoto, Japan), and the cDNA was stored at −20 °C.

### 2.5. Cloning of TcCYP6K1 and TcCYP9F2 Genes

The ORF sequences of *TcCYP6K1* and *TcCYP9F2* were amplified using specific primers designed with Primer 6.0 software (Table 1). The synthesized cDNA served as the template, and the target fragments were amplified using the Ex Taq (Code No. RR001A, Takara, Kyoto, Japan). Gel electrophoresis was then employed to detect the target fragments, followed by gel purification using the US Everbright DNA Gel Extraction Kit (UE-GX-50, UE, Jiangsu, China). The quality and concentration of the purified DNA were assessed using a NanoDrop 2000 spectrophotometer before storage at −20 °C. Subsequently, 3 μL of the purified product was mixed with 3.5 μL of Solution I and 0.5 μL of pMD18-T Vector Cloning Kit (Code No. 6011, Takara, Kyoto, Japan), centrifuged, and incubated at 16 °C for 30 min to obtain the connecting solution. Utilizing DH5α receptive cells, plasmid transformation was carried out, and the evenly growing colonies with smooth edges and transparent colors on the Petri dish were picked and dissolved in 30 μL of sterilized water; then, they were used as a colony PCR template to confirm successful cloning. After PCR amplification, 1% agarose gel electrophoresis was used to detect the correctness of the target fragment of the colony amplified using PCR. Positive clones were cultured overnight in an LB liquid medium (containing Amp) at 37 °C at 250 rpm on a shaking table, and then the bacterial culture liquid was taken and sent to a sequencing company in Chongqing for sequencing. The remaining sequenced bacterial culture liquid was mixed with 600 μL of 50% glycerol and stored at −20 °C. The Green fluorescent protein gene (GFP) was cloned by using the pMD18-T plasmid with the GFP sequence as the template, following the same method.

### 2.6. Synthesis and Injection of Double-Stranded RNA (dsDNA)

Using Primer 6.0 software, dsRNA primers (Table 1) were designed for PCR amplification. The amplified products were subjected to T cloning, and then primers with a T7 promoter were used for a cross-PCR reaction (Table 1). The dsRNA was then synthesized using the T7 RiboMax Express RNAi System kit (REF P1700, Promega, Madison, WI, USA). The integrity of total RNA was assessed by agarose gel electrophoresis, while the concentration of synthesized dsRNA was measured using NanoDrop™ 2000. The dsGFP was synthesized as a control using the same method. The synthesized dsRNAs were stored at −80 °C.

*T. castaneum* larvae at 8th-instar nymphs were immobilized on ice and injected using a Transferman 4r microinjector (Eppendorf, Hamburg, Germany) with ds*TcCYP6K1* or ds*TcCYP9F2* between the second and third abdominal segments where the epidermis is thinner. Each larva was injected with 100 nl of dsRNA (2000 ng/μL). The control group received an injection of ds*GFP*. Following injection, the larvae were divided into different groups, placed in rearing bottles, and exposed to either normal oxygen or high-CO_2_ conditions (75% CO_2_ + 25% air) with three replicates per group, each consisting of 40 larvae. After 48 h, samples were collected and stored at −80 °C for a subsequent analysis of gene expression levels, enzyme activities, and substance contents.

### 2.7. Quantitative Real-Time Polymerase Chain Reaction (qRT-PCR)

After 48 h post-injection, the survival rates of the experimental insects fed with a mixture of dsRNA and CO_2_ in each group were observed and recorded. Simultaneously, the expression levels of *TcCYP6K1*, *TcCYP9F2*, and genes related to the trehalose metabolic pathway in the surviving larvae’s bodies were measured post-injection at 48 h. Utilizing Primer 6.0 software, quantitative PCR primers were designed based on the coding region sequences of known genes related to the trehalose metabolic pathway in *T. castaneum*. The amplification of *Ribosomal Protein L13a* (*RPL13a*) was used as an internal control (Table 1). Related genes, which are included in Table 1, were expressed by TB Green Premix Ex Taq II (Tli RNaseH Plus) (Code No. RR420A, Takara, Kyoto, Japan) and qRT-PCR. The Bio-rad CFX96 Real-Time PCR Detection System (Bio-RAD Laboratories Inc., Hercules, CA, USA) was used for detection. Each PCR was performed in a final 20 μL volume, 1 μL of cDNA, 1 μL (10 μM) of each primer, 7 μL of RNase Free ddH_2_O, and 10 μL of an SYBR Green master mix. The reaction conditions were as follows: 5 s at 95 °C after 2 min at 95 °C; 59 s at 30 °C; and after 39 cycles, a melt curve analysis was conducted at 65–95 °C. The obtained data were analyzed by the 2^−ΔΔCT^ method [42].

### 2.8. Determination of Carbohydrate Content and Trehalase Activity

Twenty test insects were taken from the experimental group and the control group, and placed in PCR tubes. For each treatment, 60 larvae of *T. castaneum* were utilized for each of the 3 biological replicates. Firstly, 200 μL of PBS was added to the homogenated sample and the sample was sonicated for 30 min; later on, 800 μL of PBS was added to the crushed sample at 4 °C, 1000× *g*, and the sample was centrifuged for 20 min. After centrifugation, 350 μL of the supernatant was used to determine protein concentration, glycogen, and trehalose content, and 350 μL of the supernatant underwent ultracentrifugation at 20,800× *g* for 60 min. After ultracentrifugation, 300 μL of the supernatant was used to determine glucose concentration, trehalase activity, and protein content. We suspended the supercentrifuge precipitation in 300 μL of added PBS to determine glucose concentration, trehalase activity, and protein concentration. Then, we combined a mixture of the supernatant and suspension (60 μL), 75 μL of 40 mM trehalose (CAS 99-20-7, Sigma Aldrich, Saint Louis, MO, USA), and 165 μL of PBS, which was incubated at 37 °C for 60 min and inactivated by 5 min incubation at 100 °C. The trehalase activity was measured using the Glucose (Go) Assay Kit (Lot No. SLCD8160, Sigma, MO, USA), and the reaction was terminated by adding 12 N H_2_SO_4_ (260 μL, CAS 7664-93-9). Finally, the absorbance value was measured at 540 nm using a microplate reader (Thermo Fisher Scientific, Waltham, MA, USA). The protein contents of samples were determined using the BCA Protein Assay Kit (Cat No. P0006, Beyotime, Shanghai, China). Lastly, the anthrone method was used for the determination of the content of trehalose [43,44,45].

### 2.9. Determination of ATP Content

After washing the *T. castaneum* samples with physiological saline, 10% of test insects’ tissues were homogenized in double-distilled water. The homogenate was then placed in a boiling water bath for 10 min, followed by vigorous mixing on a vortex oscillator for 1 min at 3500 rpm. The mixture was centrifuged for 10 min, and the supernatant was used for ATP content determination using an ATP test kit (A095-1-1, Nanjing Jiancheng Bioengineering, Nanjing, China) following the manufacturer’s instructions. Absorbance values at 636 nm were measured, and ATP content was calculated using a specific formula.

### 2.10. Data Analysis

The data analysis was performed using SPSS 23.0 software, with experimental results presented as the mean ± standard error. A temporal and spatial expression analysis was conducted using one-way ANOVA for significance testing, where different letters on bars denote significant differences between groups (*p* < 0.05, ANOVA). The experiment involved three biological replicates, and significant differences between two groups were assessed using a Student’s *t*-test, with *p* (*) < 0.05 indicating significance, *p* (**) < 0.01 indicating high significance, and *p* (***) < 0.001 indicating very high significance.

## 3. Results

### 3.1. Sequence Analysis of Two P450 Genes in T. castaneum

**Figure 1:**
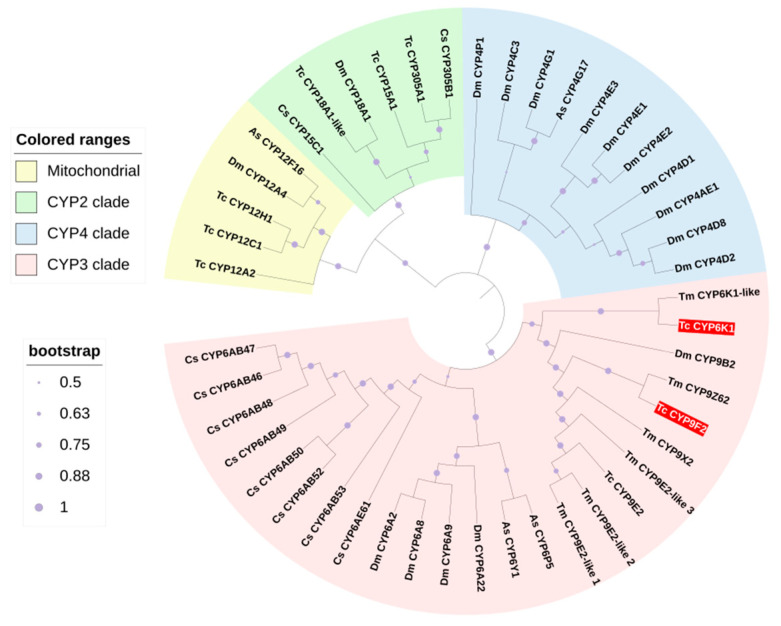
The phylogenetic tree of *TcCYP6K1* and *TcCYP9F2* in *T. castaneum*. (marked in red) The tree was conducted with MEGA 11.0 based on the amino acid sequences using the maximum likelihood method based on the Poisson model. The bootstrap replicates were 1000 in number. Numbers at each branch point represent the bootstrap values. Different background colors indicate CYP mitochondrial (yellow), CYP2 clade (green), CYP4 clade (blue), and CYP3 clade (pink). Species abbreviations are as follows: Cs, *Chilo suppressalis*; As, *Anopheles sinensis*; Tc, *Tribolium castaneum*; Tm, *Tribolium madens*.

The amino acid sequences of two P450 genes, *TcCYP6K1* (XP_015833657.1) and *TcCYP9F2* (NP_001127706.1), were obtained from the GenBank database. The encoded proteins have predicted molecular weights of 57.23 and 58.75 kDa; have theoretical pI values of 8.93 and 7.97 (Table S1), respectively; lack signal peptides; and are hydrophilic (Figure S3). Both *TcCYP6K1* and *TcCYP9F2* proteins share numerous conserved motifs (Figure S1). While *TcCYP9F2* has a transmembrane structure, *TcCYP6K1* does not (Figure S2). Both *TcCYP6K1* and *TcCYP9F2* possess the p450 conserved domain (Figure S4). A phylogenetic analysis revealed that *TcCYP6K1* and *TcCYP9F2* of *T. castaneum* exhibit the highest homology with *TmCYP6K1-like* and *TmCYP9Z62*, respectively, all belonging to the CYP3 group (Figure 1).

### 3.2. Temporal and Spatial Expression Pattern of Two P450 Genes in T. castaneum

**Figure 2:**
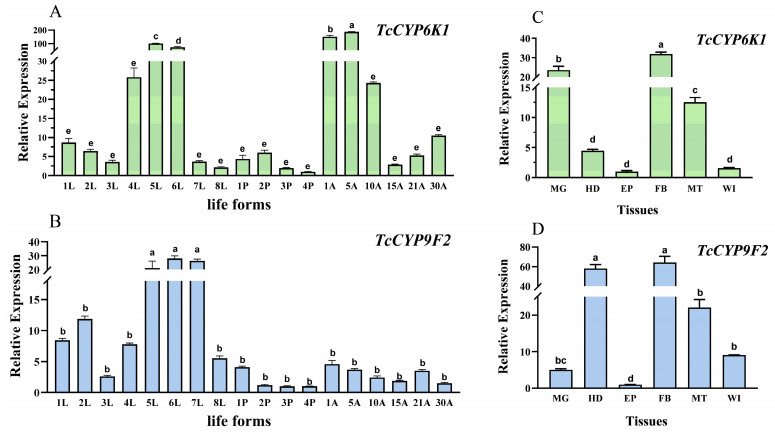
Relative expression levels of *TcCYP6K1* and *TcCYP9F2* in different life forms and tissues in *T. castaneum*. (**A**) Relative expression levels of *TcCYP6K1* in different life forms. (**B**) Relative expression levels of *TcCYP9F2* in different life forms. (**C**) Relative expression levels of *TcCYP6K1* in different tissues. (**D**) Relative expression levels of *TcCYP9F2* in different tissues. 1L-8L, larvae at 1–8 instars; 1P-4P, pupae for 1–4 days; 1A, adult at 1st day; 5A, adult at 5th day; 10A, adult at 10th day; 15A, adult at 15th day; 21A, adult at 21st day; 30A, adult at 30th day; MG, midgut; HD, head; EP, epidermis; FB, fatty body; MT, malpighian tube; WI, wing. Three biological replicates were conducted for each development stage, and 35 test insects were collected for each replicate. Each replicate involved the dissection of 100 adult insects (mean ± SE; Tukey’s test; different letters in the figure indicate significant differences between groups, *p* < 0.05).

The *TcCYP6K1* gene is highly expressed during both larval and adult stages, while *TcCYP9F2* shows high expression only in fifth- to seventh-instar larvae. The relative expression levels of both genes during the pupal stage are significantly lower than those during the larval and adult stages (Figure 2A,B). *TcCYP6K1* peaks in expression on day 5 of the adult stage, being 186.4 times higher than on day 4 of the pupal stage (Figure 2A).

The expression levels of the *TcCYP6K1* and *TcCYP9F2* genes are the lowest in the epidermis (Figure 2C,D). The *TcCYP6K1* gene is predominantly expressed in the fatty body, midgut, and malpighian tube, 31.9, 23.5, and 12.5 times higher than in the epidermis (Figure 2C). The *TcCYP9F2* gene is mainly expressed in the fatty body, head, and malpighian tube, 64.1, 58.0, and 22.0 times higher than in the epidermis, respectively (Figure 2D).

### 3.3. Detection of Silencing Efficiency and Survival Rate after CO_2_ Stress

**Figure 3:**
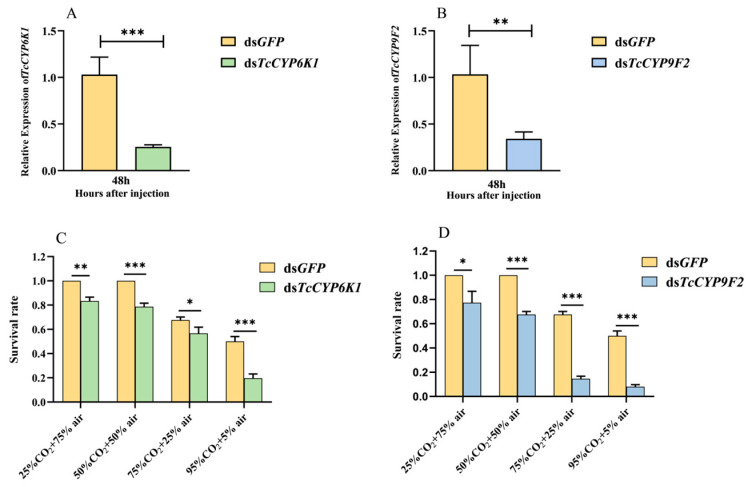
The dsRNA interference efficiency and CYP gene detection of changes in its sensitivity to CO_2_. (**A**) ds*TcCYP6K1* interference efficiency; (**B**) ds*TcCYP9F2* interference efficiency; (**C**) the *TcCYP6K1* detection of changes in its sensitivity to CO_2_; (**D**) the Tc*CYP9F2* detection of changes in its sensitivity to CO_2_. Values are presented as the means ± SE. ***: *p* < 0.001, **: *p* < 0.01, *: *p* < 0.05 (independent samples *t*-test). Three biological replicates were performed on 60 *T. castaneum* larvae in each treatment.

The relative expression levels of *TcCYP6K1* and *TcCYP9F2* were detected through qRT-PCR after the injection of dsRNA and 48 h incubation. The results indicate that compared to the control group, the relative expression levels of *TcCYP6K1* and *TcCYP9F2* genes decreased by 77.6% and 70.2%, respectively (Figure 3A,B), indicating that RNAi successfully inhibited gene expression and could be used in subsequent experiments.

After 48 h of dsRNA interference injection, survival rates of test insects were assessed under different concentrations of CO_2_ treatment (25%, 50%, 75%, and 95%) and statistically analyzed. The results revealed that under different concentrations of CO_2_ atmospheres, the survival rates of the ds*TcCYP6K1* and ds*TcCYP9F2* treatment groups were significantly lower than the ds*GFP* control group (Figure 3C,D). Particularly at the 95% CO_2_ concentration, compared to the control group, the survival rate decreased by 30.3% in the ds*TcCYP6K1* treatment group (Figure 3C) and by 42.0% in the ds*TcCYP9F2* treatment group (Figure 3D).

### 3.4. Effect on Carbohydrate Metabolism after Silencing and under CO_2_ Stress

**Figure 4:**
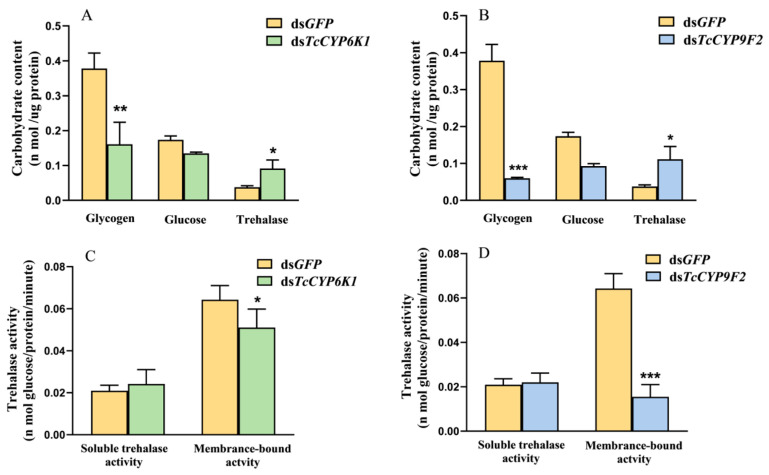
Effects of CYP gene RNAi on the contents of carbohydrates and trehalase activity in the 75% CO_2_ condition. (**A**) Effects of Tc*CYP6K1* RNAi on the contents of carbohydrates with the 75% CO_2_ condition. (**B**) Effects of Tc*CYP9F2* RNAi on the contents of carbohydrates with the 75% CO_2_ condition. (**C**) Effects of Tc*CYP6K1* RNAi on the trehalase activity with the 75% CO_2_ condition. (**D**) Effects of Tc*CYP9F2* RNAi on the trehalase activity with the 75% CO_2_ condition. For each treatment, 60 larvae of *T. castaneum* were taken for 3 biological replicates. Values are presented as the means ± SE. ***: *p* < 0.001, **: *p* < 0.01, *: *p* < 0.05 (independent samples *t*-test).

The experimental groups injected with ds*TcCYP6K1* and ds*TcCYP9F2*, and the control group injected with ds*GFP*, were treated with 75% CO_2_. It was found that compared to the control group, the glycogen content of the larvae in the experimental groups significantly decreased, while glucose content did not show significant changes. However, there was a significant increase in the trehalose content compared to the control group (Figure 4A,B).

Results from trehalase activity assays showed that the soluble trehalase activity of the larvae in the experimental groups treated with 75% CO_2_ did not show significant changes compared to the control group, while the membrane-bound trehalase activity significantly decreased (Figure 4C,D).

### 3.5. Effect on ATP Content after Silencing and under CO_2_ Stress 

**Figure 5:**
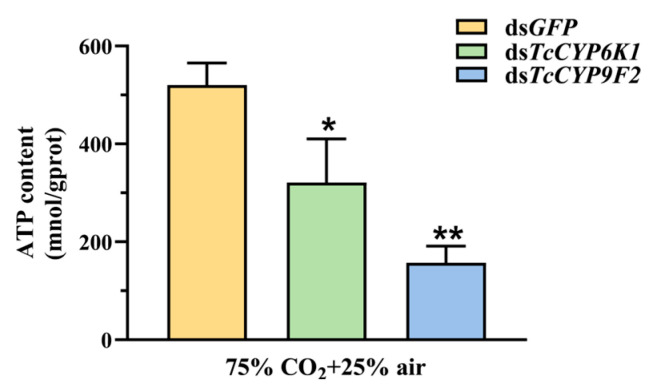
The effect of CYP gene RNAi on ATP content in *T. castaneum* with the 75% CO_2_ condition. Values are presented as the means ± SE. **: *p* < 0.01, *: *p* < 0.05 (independent samples *t*-test). Three biological replicates were performed on 60 larvae of *T. castaneum* in each treatment.

After silencing the genes *GFP*, *TcCYP6K1*, and *TcCYP9F2*, we treated the test insects with 75% CO_2_ and measured ATP levels. The results showed a significant decrease in ATP levels when interfering with *TcCYP6K1* and *TcCYP9F2* compared to ds*GFP* injection. Particularly, the injection of ds*TcCYP9F2* led to the most significant drop in larval ATP levels, reaching as low as 363 nmol/gprot (Figure 5).

### 3.6. Effect on Trehalose Metabolism Pathway after Silencing and under CO_2_ Stress 

**Figure 6:**
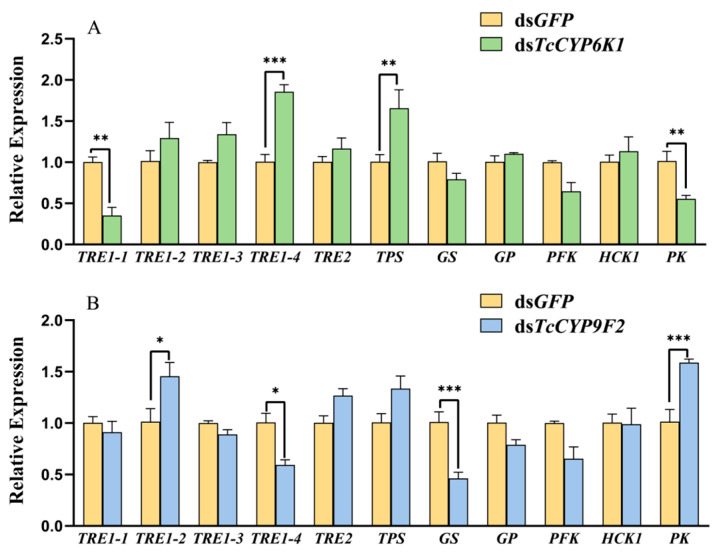
The effect of *TcCYP6K1* (**A**) and *TcCYP9F2* (**B**) RNAi on the expression of trehalose metabolic pathway-related genes in *T. castaneum* under the 75% CO_2_ condition. TRE, trehalase; TPS, trehalose-6-phosphate synthase; GS, glycogen synthase; GP, glycogen phosphorylase; PFK, phosphofructokinase; HCK1, hexokinase; PK, pyruvate kinase. Three biological replicates were performed on 60 larvae of *T. castaneum* in each treatment. Values are presented as the means ± SE. ***: *p* < 0.001, **: *p* < 0.01, *: *p* < 0.05 (independent-samples *t*-test).

In the experimental group of eighth-instar larvae of *T. castaneum*, ds*TcCYP6K1* and ds*TcCYP9F2* were injected, while the control group was injected with ds*GFP*. After 48 h, 75% CO_2_ treatment was applied. Gene expression related to the trehalose metabolism pathway was examined. In comparison to the control group, the injection of ds*TcCYP6K1* resulted in a significantly reduced expression of *TRE1-1* and *PK* genes, and a significantly increased expression of *TRE1-4* and *TPS* genes. The expression of other genes did not show significant changes (Figure 6A). In the experimental group injected with ds*TcCYP9F2*, a significant downregulation of *TRE1-4* and *GS* genes and significant upregulation of *TRE1-2* and *PK* genes were observed compared to the control group, while the expression of other genes remained unchanged (Figure 6B).

## 4. Discussion

CYPs are a class of detoxification enzymes widely present in aerobic organisms [46], primarily involved in the metabolism of endogenous substances and the detoxification of exogenous toxic compounds. The expression pattern of the P450 gene is significantly different in different tissues and life forms, which provides clues for exploring its physiological function [47,48,49,50]. In this study, we used qRT-PCR to analyze the temporal and spatial expression of *TcCYP6K1* and *TcCYP9F2* genes. In developmental expression, we found that the *TcCYP6K1* gene was highly expressed in late larval and adult stages, and *TcCYP9F2* was only highly expressed in late larval stages (Figure 2A,B). Similarly, in *Bactrocera minax*, the *CYP314A1* gene displayed higher expression levels during the larval stage and lower levels during the pupal stage [51]. Furthermore, in *T. castaneum*, *CYP4Q4*, *CYP4G7*, *CYP4BR3*, and *CYP345A1* are most commonly expressed in larvae and mature adults [52]. In terms of gene expression patterns, the expression sites of the *TcCYP6K1* gene are primarily concentrated in the fatty body, midgut, and malpighian tubules (Figure 2C). Similarly, the expression sites of the *TcCYP9F2* gene are predominantly in the fatty body, head, and malpighian tubules (Figure 2D). These tissues have been recognized as crucial detoxification organs, with some detoxification-related P450 genes exhibiting high expression levels in these locations [53,54,55]. For example, in *Drosophila melanogaster*, *CYP311A1* is localized at the anterior midgut [56]. In *T. castaneum*, Tc*CYP4BN6* and Tc*CYP6BQ11* have been detected to express in the malpighian tubules [57]. Additionally, in *Helicoverpa armigera*, *CYP6AB12*, *CYP321B1*, *CYP6AB60*, and *CYP321A19* are significantly expressed in the midgut and fatty body [58]. Moreover, in *Plutella xylostella*, the *CYP367* gene shows high expression in the head with the ability to detoxify [59]. Consequently, it can be inferred that the *TcCYP6K1* and *TcCYP9F2* genes in *T. castaneum* possess detoxification abilities, primarily functioning during the larval stage.

To further understand the detoxification role of *TcCYP6K1* and *TcCYP9F2* genes in *T. castaneum* under the CO_2_ atmosphere, we injected eighth-instar larvae with dsRNA and subjected them to CO_2_ treatment [60,61,62]. The results revealed that under different concentrations of CO_2_ treatment, the survival rates of the ds*TcCYP6K1*- and ds*TcCYP9F2*-treated groups were significantly lower than those of the control group (Figure 3C,D). Similarly, the essential oil of Artemisia vulgaris (EOAV) significantly induced the expression of the *TcCYP6BQ7* gene in *T. castaneum*, and silencing this gene increased larval mortality in response to EOAV from 49.67% to 71.67% [50]. Moreover, dichlorvos significantly induced the expression of *TcCYP4BN6* and *TcCYP6BQ11* in *T. castaneum*, and silencing these genes led to a significant increase in larval mortality upon insecticidal treatment [57]. Furthermore, terpinen-4-ol significantly induced the expression of *TcCYP9Z6* in *T. castaneum*, and RNAi-mediated silencing of this gene resulted in an increase in larval mortality from 47.75% to 63.92% [63]. Therefore, it is inferred that *TcCYP6K1* and *TcCYP9F2* are key genes and play essential roles in *T. castaneum* responding to CO_2_ stress. The downregulation of *TcCYP6K1* and *TcCYP9F2* gene expression increases the sensitivity of *T. castaneum* to CO_2_, inhibiting aerobic metabolism and disrupting their life activities (Figure 3C,D). However, the involvement of *TcCYP6K1* and *TcCYP9F2* genes in the detoxification mechanism of endogenous metabolites under high-CO_2_ conditions remains unclear and requires further investigation.

In the larva of *T. castaneum*, silencing of the *TcCYP6K1* and *TcCYP9F2* genes resulted in a suppression of aerobic metabolism in *T. castaneum* exposed to high CO_2_ in a closed environment with high CO_2_ and low O_2_ levels, leading to the consumption of energy in the form of ATP [17]. ATP, the fundamental unit providing energy, is the primary source of energy for most cellular activities [64]. The ATP content in *T. castaneum* significantly decreased (Figure 5), weakening its energy metabolism to a certain extent. Glycogen, an essential metabolic and energy storage substance, synthesized and stored mainly in fatty bodies, can rapidly convert into trehalose and glucose to provide energy to other tissues when insects require an energy supply [65,66]. In this study, the glycogen content significantly decreased in *T. castaneum* larvae treated with dsRNA injection and 75% CO_2_ exposure, with no significant change in glucose content and a significant increase in trehalose content (Figure 4A,B). It is inferred that glycogen is converted into glucose, partially compensating for the deficiency in carbohydrate metabolism and energy supply [17]. Additionally, trehalose, as a vital sugar and energy source in the insect hemolymph, plays a crucial role in various physiological activities in insect development and stress resistance [67,68,69,70]. Studies have shown that *Polypedilum vanderplanki* can effectively accumulate trehalose under dry stress conditions to help insects cope with desiccation stress [71]. 

Trehalose is synthesized in the fatty body mainly by trehalose-6-phosphate synthase (TPS) and trehalose-6-phosphate phosphatase (TPP) [72]. Subsequently, trehalose is released into the lymphatic system and transported to various tissues through the lymphatic circulation to exert its functions [69]. Trehalase enzymes are categorized into soluble trehalase (TRE1) and membrane-bound trehalase (TRE2). Following treatment of larvae with silenced *TcCYP6K1* and *TcCYP9F2* genes under high-CO_2_ conditions, a significant decrease in trehalase activity was observed (Figure 4C,D). Moreover, there was a marked upregulation in *TPS* gene expression and downregulation in *TRE* gene expression (Figure 6). Similarly, in fruit flies, inducing the overexpression of *TPS* to elevate trehalose levels enhanced the flies’ hypoxia tolerance [73]. Consequently, we can infer that silencing of *TcCYP6K1* and *TcCYP9F2* genes hinders trehalose metabolism in *T. castaneum*, leading to insufficiency of trehalase enzymes for trehalose breakdown and ultimately causing insect mortality due to the inability to maintain normal physiological activities. The distinct and intricate potential functions of the carbohydrate metabolic pathway necessitate further in-depth exploration. These results indicate that CYP genes may impact the resistance of *T. castaneum* to hypoxia by modulating the carbohydrate metabolic pathway through synthesis or breakdown processes.

## 5. Conclusions

In this paper, the dsRNA combined with high-CO_2_ controlled atmosphere treatment was used to silence the *TcCYP6K1* and *TcCYP9F2* in *T. castaneum*, to investigate the role of P450 genes influencing in the trehalose metabolic pathway under high CO_2_ stress. Reducing the expression of *TcCYP6K1* and *TcCYP9F2* enhances the sensitivity of the red flour beetle to CO_2_, demonstrating that *TcCYP6K1* and *TcCYP9F2* are key factors in the response to high levels of CO_2_ in *T. castaneum.* The experimental results show a significant increase in trehalose content, a significant decrease in trehalose synthase activity, a significant upregulation of *TPS* gene expression, and a significant downregulation of *TRE* gene expression. It is speculated that the silencing of the *TcCYP6K1* and *TcCYP9F2* genes hinders the metabolism of trehalose in *T. castaneum*, leading to insufficient trehalase enzymes or trehalose breakdown, thereby disrupting normal physiological activities and resulting in death. Therefore, CYP genes may impact the CO_2_ resistance in *T. castaneum* by influencing in the synthesis or decomposition of carbohydrate metabolic pathways. This provides a theoretical basis for the utilization of novel nucleic acid pesticides in low-oxygen grain storage technology.

## Figures and Tables

**Table 1:** Primers used in the dsRNA synthesis and qRT-PCR detection.

Gene	Forward Primer (5′-3′)	Reverse Primer (5′-3′)	Primer Use
ds*TcCYP9F2*	CCTACAAATACTGGACCGA	CAGGAAGTACCCCAACAA	Amplification of dsRNA
ds*TcCYP6K1*	AAGAAGCGGAGTGGTGGA	GCGAAGTAATTGTTATGGAGG	
ds*GFP*	AAGGGCGAGGAGCTGTTCACCG	CAGCAGGACCATGTGATCGCGC	
T7 promoter	GGATCCTAATACGACTCACTATAGG		
*TcRPL13a*	ACCATATGACCGCAGGAAAC	GGTGAATGGAGCCACTTGTT	qRT-PCR analysis
*TcCYP9F2*	ACCGGCTACCAAGAATCCAA	GTGACCTTTCCGTTGCAGTT	
*TcCYP6K1*	AACCCCTTACGTTGGCATCT	GCCAGTTGTCGTTCTTTGCA	
*TRE1-1*	AACCAAACACTCACTCATTCC	AATCCAATAAGTGTCCCAGTAG	
*TRE1-2*	GAAGTATCGGTTGGCTCG	GAGTGGGGTTGATTGTGC	
*TRE1-3*	CTTGAACGCCTTCCTCTG	CCATCCTCGTGGTCATAAA	
*TRE1-4*	CTACCTAAACCGCTCCCA	TGTCCAGCCAGTACCTCAG	
*TRE2*	TGTTGTGCGTTTGTGCTC	GGACGGCTTATTGTTGTTTA	
*TPS*	GATTCGCTACATTTACGGG	GAACGGAGACACTATGAGGAC	
*GS*	ATTGGAGGAGTCTAGGAGTGTAC	CCGAATCGCTTTCATCAT	
*GP*	CCGATGGCTCCTTATGTG	GTATGCGTTTGACGTGGAT	
*PFK*	CTACGAAAATGTCCGAAGG	GTTGCGGTCAAAAGGTGT	
*HCK1*	GAGGTATGTCTGCGAATGC	TGGAAATGTGGGTGGAAC	
*PK*	CAACCGACGAAAAGTATGC	TTCACCCCTTTACTACTCCC	

## Data Availability

The data presented in this study are available on request.

## Supplementary Materials

The following supporting information can be downloaded at: https://www.mdpi.com/article/10.3390/insects15070502/s1, Table S1: Physicochemical properties of *TcCYP6K1* and *TcCYP9F2.* Figure S1: conserved motif of *TcCYP6K1* and *TcCYP9F2.* Figure S2: Transmembrane domain of *TcCYP6K1* and *TcCYP9F2*. Figure S3: *TcCYP6K1* and *TcCYP9F2* signal peptide. Figure S4: Conserved Domains of *TcCYP6K1* and *TcCYP9F2*.
