# Supplementary material for: The Role of TcCYP6K1 and TcCYP9F2 Influences Trehalose Metabolism under High-CO2 Stress in Tribolium castaneum (Coleoptera)
Source: Insects. 2024 Jul 5;15(7):502. doi: 10.3390/insects15070502 (PMC11276637; doi:10.3390/insects15070502)
Supplement: Supplementary file 1 [file insects-15-00502-s001.zip › insects-3032867-supplementary.pdf]

Supplement:

Table S1. Physicochemical properties of *TcCYP6K1* and *TcCYP9F2*.

Physicochemical parameters	*TcCYP6K1*	*TcCYP9F2*
Formula	$C_{2628}H_{4066}N_{658}O_{726}S_{23}$	$C_{2686}H_{4122}N_{678}O_{754}S_{24}$
Number of amino acids	57.23 kDa	58.75 kDa
Molecular weight	8.93	7.97
Theoretical pI	58	63
Total number of negatively charged residues	68	65
Total number of positively charged residues	88.35	78.29
Aliphatic index	33.81	36.74
Instability index	-0.105	-0.355
Presence or absence of signal peptides	0.016<0. 5	0.2653<0.5
Phosphorylation site	T23/S34/Y11	T22/S31/Y11
Subcelluar location	peroxisome	Endoplasmic reticulum

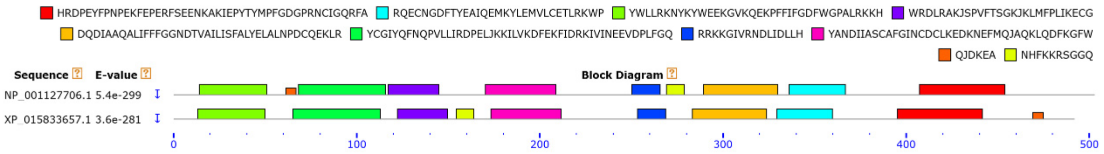

Figure S1. conserved motif of *TcCYP6K1* and *TcCYP9F2.*

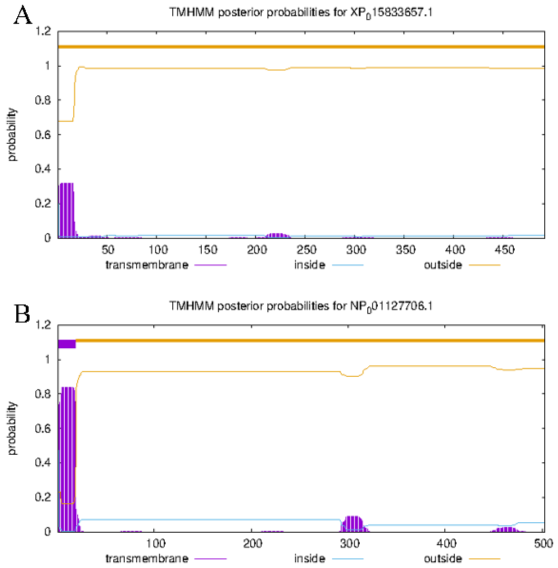

Figure S2. Transmembrane domain of *TcCYP6K1* (A) and *TcCYP9F2* (B). The transmembrane structures of TcCYP6K1 and TcCYP9F2 proteins were predicted using the TMHMM-2.0. TcCYP6K1 protein lacks a transmembrane structure. The TcCYP9F2 protein has a transmembrane structure.

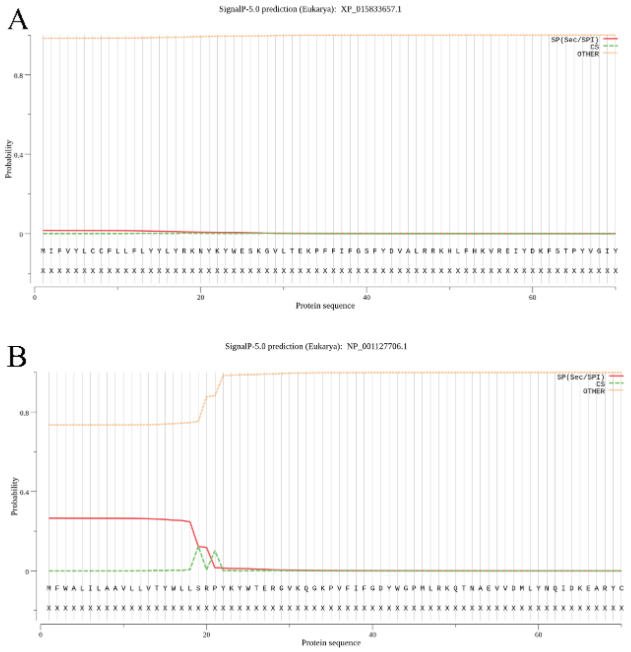

Figure S3. *TcCYP6K1* (A) and *TcCYP9F2* (B) signal peptide. Signal peptides were predicted using SignalP-5.0, and both TcCYP6K1 and TcCYP9F2 proteins had signal peptide prediction probabilities less than 0.5, indicating the absence of signal peptides.

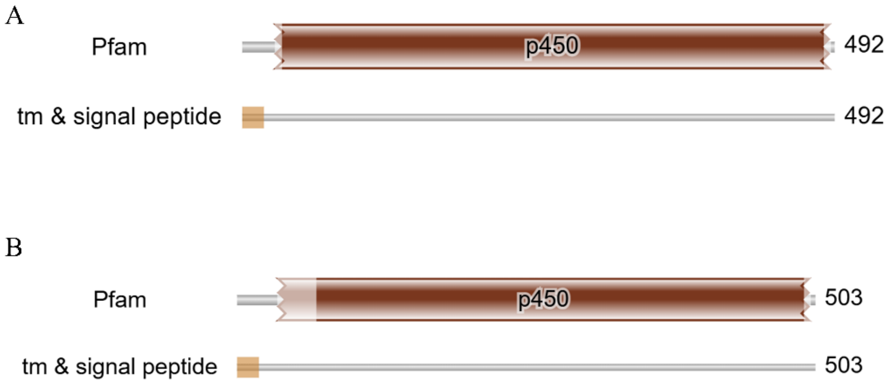

Figure S4. Conserved Domains of *TcCYP6K1* (A) and *TcCYP9F2* (B). The protein sequences of TcCYP6K1 and TcCYP9F2 from the *Tribolium castaneum* were analyzed using the HMMER online tool. Both proteins have a single conserved domain and belong to the cytochrome P450 family.
